# Supplementary material for: The BET Protein Inhibitor JQ1 Decreases Hypoxia and Improves the Therapeutic Benefit of Anti-PD-1 in a High-Risk Neuroblastoma Mouse Model
Source: Cells. 2022 Sep 6;11(18):2783. doi: 10.3390/cells11182783 (PMC9497090; doi:10.3390/cells11182783)
Supplement: Supplementary file 1 [file cells-11-02783-s001.zip › cells-1839942-supplementary.pdf]

cells MDPI

Supplementary Data

The BET Protein Inhibitor JQ1 Decreases Hypoxia and Improves the Therapeutic Benefit of Anti-PD-1 in a High-Risk Neuroblastoma Mouse Model

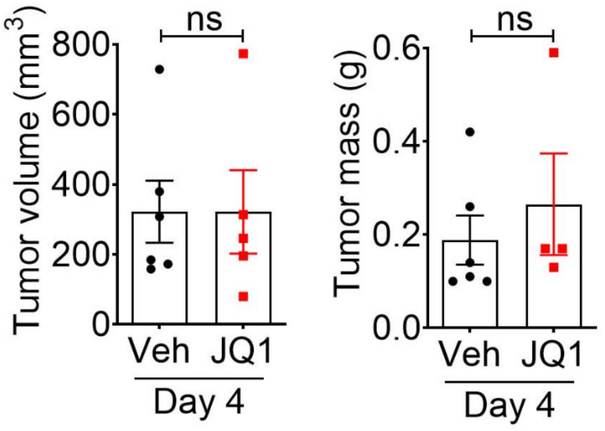

	Volume	mass (g)
Mean of Vehicle-treated tumors	321.9	0.1883
Mean of JQ1-treated tumors	321.6	0.265
Difference between means (Veh - JQ1) ± SEM	(0.2583 ± 145.7)	(-0.07667 ± 0.1082)

Supplementary Figure S1. Volumes (left) and masses (right) of TH-MYCN tumors at day 4 in mice treated with vehicle (Veh) or JQ1. Each dot represents one tumor. Results are shown as mean ± SEM (error bars). Statistically significant differences were calculated compared to the control group (veh) using an unpaired two-tailed Student's t-test (ns: not significant). The table at the bottom shows the means of tumor volume and mass in vehicle- and JQ1-treated tumors and the difference between means.

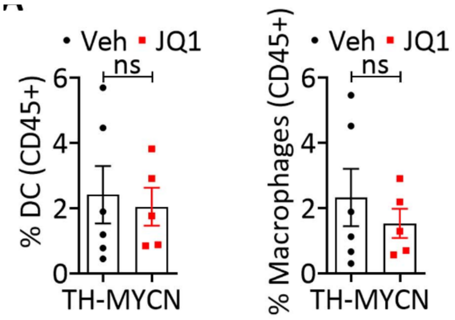

Supplementary Figure S2. Flow cytometry quantifies total DC and macrophages infiltrating vehicle-treated or JQ-1-treated TH-MYCN tumors on day 4. The defined subpopulations were gated and quantified in live CD45+ cells. Each dot represents one tumor. The data are reported as the average of six or five mice per group. Results are shown as mean ± SEM (error bars). Statistically significant differences (indicated by asterisks) are compared to vehicle-treated tumors using an unpaired two-tailed Student's t-test (ns: not significant).
